# Electrospun aligned poly(ε-caprolactone) nanofiber yarns guiding 3D organization of tendon stem/progenitor cells in tenogenic differentiation and tendon repair

**DOI:** 10.3389/fbioe.2022.960694

**Published:** 2022-08-30

**Authors:** Qiao Yang, Jianfeng Li, Weiwei Su, Liu Yu, Ting Li, Yongdi Wang, Kairui Zhang, Yaobin Wu, Ling Wang

**Affiliations:** ^1^ Biomaterials Research Center, School of Biomedical Engineering, Southern Medical University, Guangzhou, China; ^2^ The First School of Clinical Medicine, Southern Medical University, Guangzhou, China; ^3^ Guangdong Engineering Research Center for Translation of Medical 3D Printing Application, Guangdong Provincial Key Laboratory of Medical Biomechanics, National Key Discipline of Human Anatomy, School of Basic Medical Sciences, Southern Medical University, Guangzhou, China; ^4^ Division of Orthopaedics and Traumatology, Department of Orthopaedics, Nanfang Hospital, Southern Medical University, Guangzhou, China

**Keywords:** electrospinning, nanofiber yarns, 3D alignment, tendon stem/progenitor cells, tenogenic differentiation, tendon repair

## Abstract

Hierarchical anisotropy structure directing 3D cellular orientation plays a crucial role in designing tendon tissue engineering scaffolds. Despite recent development of fabrication technologies for controlling cellular organization and design of scaffolds that mimic the anisotropic structure of native tendon tissue, improvement of tenogenic differentiation remains challenging. Herein, we present 3D aligned poly (ε-caprolactone) nanofiber yarns (NFYs) of varying diameter, fabricated using a dry-wet electrospinning approach, that integrate with nano- and micro-scale structure to mimic the hierarchical structure of collagen fascicles and fibers in native tendon tissue. These aligned NFYs exhibited good *in vitro* biocompatibility, and their ability to induce 3D cellular alignment and elongation of tendon stem/progenitor cells was demonstrated. Significantly, the aligned NFYs with a diameter of 50 μm were able to promote the tenogenic differentiation of tendon stem/progenitor cells due to the integration of aligned nanofibrous structure and suitable yarn diameter. Rat tendon repair results further showed that bundled NFYs encouraged tendon repair *in vivo* by inducing neo-collagen organization and orientation. These data suggest that electrospun bundled NFYs formed by aligned nanofibers can mimic the aligned hierarchical structure of native tendon tissue, highlighting their potential as a biomimetic multi-scale scaffold for tendon tissue regeneration.

## Introduction

Partial or complete tendon ruptures caused by accidents and sports disrupt collagen alignment, leading to loss of function and reduced mobility (Hasan et al., 2014; Nau and Teuschl, 2015). However, native tendon has a poor endogenous ability to regenerate after injury due to fewer blood vessels and nerves in the tendon tissue (Silver et al., 2003; Longo et al., 2011; Kalson et al., 2015). Alternatively, tissue engineering (TE) strategies have demonstrated potential application for tendon tissue regeneration, and biomaterial scaffolds provide a temporary extracellular matrix (ECM) for cell adhesion and proliferation, which is a key element in guiding tissue regeneration (Chen et al., 2015; Zhang, 2020). Tendon has a hierarchical organization, and the subunits of the tendon are the fascicles (diameters: 100–500 μm), which consist of highly aligned collagen fibers (diameters: 1–20 μm) (Zhang et al., 2006; Carniel and Fancello, 2019; Puetzer et al., 2021). Such hierarchical structure is easily disordered upon injury due to the chaotic arrangement of neo-collagen in scar tissue appearing at the site of the defect, thereby weakening the mechanical properties and functionality of the injured tendon (Sharma and Maffulli, 2005). This critical structure-function relationship reflects the design strategy for developing a suitable scaffold to facilitate the capacity of TE tendon to mimic the hierarchical structure of native tendon. The functional recovery of tendon tissue is a critical point in tendon TE scaffold development and is an ongoing challenge.

Recently, various fabrication technologies, such as electrospinning (Wu et al., 2016; Ma et al., 2018; Zhu et al., 2021), self-assembly (Gonzalez-Masis et al., 2020; Yin et al., 2020), and 3D bioprinting (Merceron et al., 2015; Chae et al., 2021; Zhang et al., 2021), have been developed to mimic the structure of native tendon by controlling aligned cellular organization. Among these techniques, electrospinning has been widely used in tendon TE due to its ability to prepare aligned nano- and micro-fibers efficiently and mimic the anisotropic hierarchical structure of native tendon tissue (Sensini and Cristofolini, 2018). In previous studies, continuous electrospun filaments have been made through changing the type of collectors (Dabirian et al., 2007; Bosworth, 2014; Laranjeira et al., 2017; Xie et al., 2021) to prepare a hierarchical structure for tendon tissue (Wu et al., 2017b; Sensini et al., 2019; Sensini et al., 2021). Mouthuy et al. (2015) fabricated a polydioxanone filament using a wire collector and assembled filaments into yarn to mimic the structure of native tendon, but then failed to control the diameter of the filaments. To achieve a similar effect, Sensini et al. (2019) prepared a multi-scale scaffold by rolling aligned nanofiber films to form a unit, then assembled them to make the multi-scale scaffold. Similarly, Laranjeira et al. (2017) produced braided and woven scaffolds using different textile techniques to weave together nanofiber threads into fibrils, then fibers, and, ultimately, 3D scaffolds. In addition to the morphological and mechanical features of the scaffold, it is well-established that suitable diameters and functions are extremely important for cell proliferation and differentiation. However, the effects of bundled nanofiber diameter have not been investigated.

Recently, we developed a series of aligned nanofiber yarns (NFYs) based on an advanced wet-dry electrospinning technique to induce 3D cellular alignment and differentiation. We further developed biomimetic anisotropic scaffolds based on these aligned NFYs for various TE applications, including skeletal muscle, cardiac, and nerve TE (Wang et al., 2015; Wu et al., 2017b; Wang et al., 2019). However, due to the similar structure of tendon tissue, the effect of aligned NFYs on tenogenic differentiation *in vitro* and tendon repair *in vivo* has remained unclear.

Furthermore, stem cells have been regarded as a promising cell type for tendon regeneration. Among the types of stem cells isolated from different tissue sources, tendon stem/progenitor cells (TSPCs), which were isolated from tendon sources, were thought to be especially suitable for the tendon microenvironment and highly likely to differentiate into tenocytes (Guo et al., 2016). However, a recent study showed that TSPCs tend to undergo chondrogenic/osteogenic differentiation under inflammatory conditions, which ultimately leads to ectopic calcification in tendon tissue (Chen et al., 2019). It was found that local stem/progenitor cells are induced into an erroneous differentiation in the diseased tendon (Hu et al., 2016). Therefore, promoting tenogenic differentiation of TSPCs plays a key role in tendon regeneration, yet remains a challenging prospect. Although several previous studies reported that scaffolds with aligned structures could improve the tenogenic differentiation of TSPCs (Yin et al., 2010), there are still few reports on the design of aligned scaffolds that integrate with nano-, micro- and macro-scale structures and would mimic the hierarchical structure of native tendon to promote tenogenic differentiation of TSPCs and tendon repair.

**FIGURE 1:**
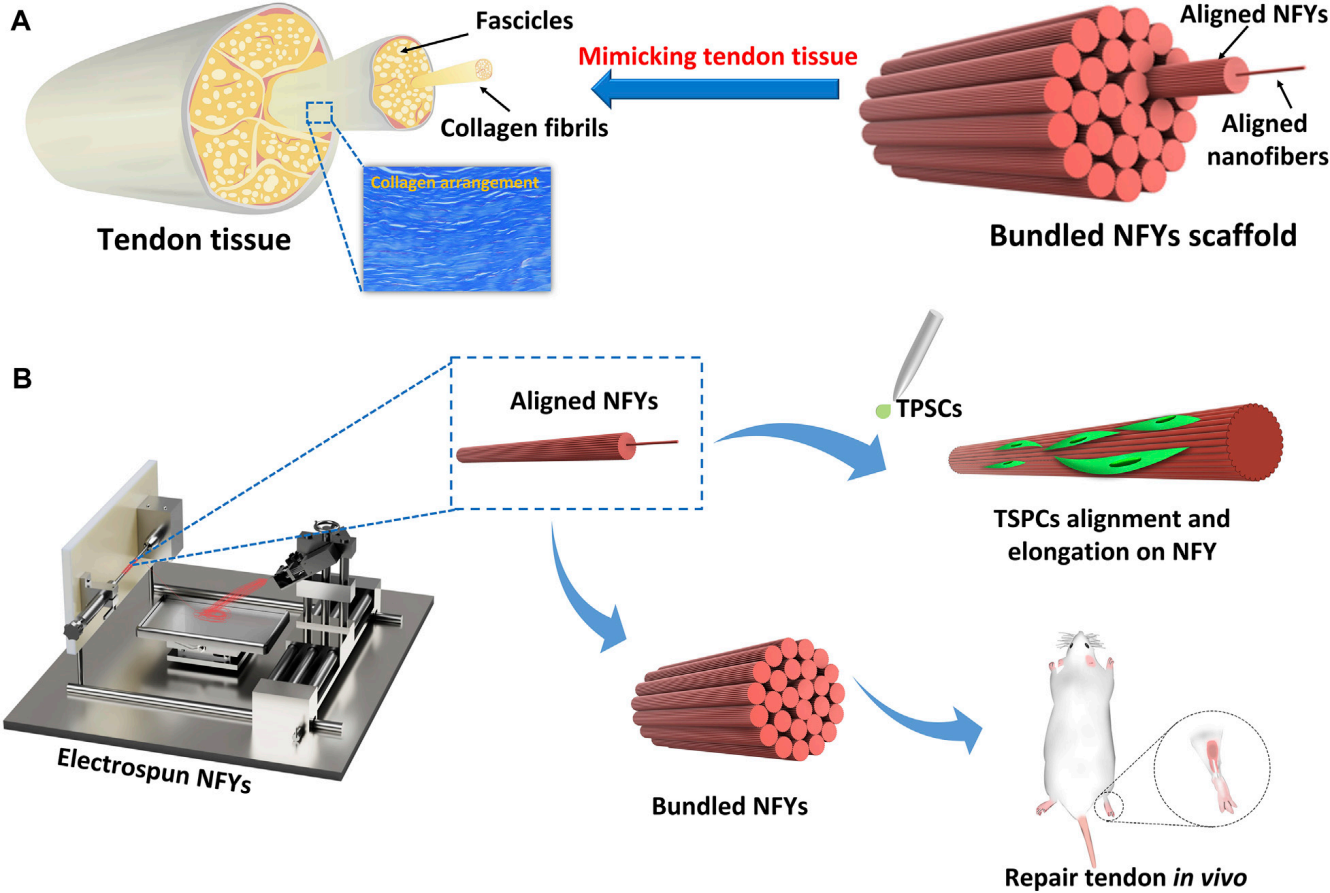
The preparation scheme of aligned PCL NFYs and their applications for tenogenic differentiation *in vitro* and tendon repair *in vivo*. **(A)** The complex hierarchical structure of native tendon tissue, consisting of aligned collagen fascicles with aligned fibers. The bundled NFY scaffold mimicked the hierarchical structure of native tendon. **(B)** Fabrication scheme based on aligned NFYs that mimic native tendon tissue to produce the hierarchical structure of scaffolds through a dry-wet electrospinning technique.

Herein, we report the development of a series of aligned bundled scaffolds based on NFYs prepared by our previously developed wet-dry electrospinning technique, which demonstrated well-defined nano/microarchitecture that mimicked the hierarchical structure of fascicles and collagen fibers in native tendon tissue (Figure 1A). TSPCs were seeded on aligned NFYs, and the effects of the nano- and micro-structure of aligned NFYs on the cellular alignment and organization of TSPCs were investigated. After culture for 7 days in differentiation medium, the effects of different NFY diameters on the tenogenic differentiation of TSPCs was also investigated. Furthermore, aligned NFY bundle scaffolds were implanted into the rat tendon defect model to investigate their ability to improve *in vivo* tendon repair. The electrospun aligned NFYs were able to guide cellular alignment and organization, thereby improving tenogenic differentiation of TSPCs *in vitro*, which suggests that these aligned NFY bundle scaffolds mimic the hierarchical structure of collagen fascicles and fibers in native tendon tissue and demonstrates great potential for tendon TE applications.

## Materials and methods

### Materials

Poly (ε-caprolactone) (PCL, average Mn = 80 000), collagenase type I (collagenase from *Clostridium histolyticum* for general use), Poly-L-lysine (PLL), paraformaldehyde (PFA), 2-phospho-L-ascorbic acid trisodium, bovine serum albumin (BSA), and pentobarbital were obtained by Sigma-Aldrich (Sigma-Aldrich, Saint Louis, United Kingdom). Hexafluoroisopropanol (HFIP) was purchased from Macklin (Macklin, China).

### Preparation of PCL aligned nanofiber yarns

The aligned PCL NFYs were prepared via dry-wet electrospinning following our previously reported process (Wang et al., 2015; Wu et al., 2017b; Wang et al., 2019). PCL was dissolved at 100 mg/ml in hexafluoroisopropanol at room temperature for 12 h. The schematic diagram in Figure 1 illustrates the working principle of the electrospinning device (ET-2535, Ucalery, China). In brief, the PCL solution was drawn into a 5 ml syringe with a 21G needle with a length of 15 mm, an inner diameter of 0.5 mm, and an outer diameter of 0.8 mm (Ucalery, China). Injection speed was 1 ml/h using a syringe pump (ET-2535, Ucalery, China). The length of the drum was 21 cm and the diameter was 3 cm. Spinning was induced by adjusting the positive and negative pressure of the electrospinning instrument; the positive voltage was set at 18 kV and the negative voltage at 2 kV. During the electrospinning process, the random nanofibrous mat was received on the surface of a water/ethanol (V/V 8:2) mixture, and the continuous NFYs were then obtained by dragging from the surface of the solution using a rolling receptor to gain. The distance between the needle and surface of the solution was approximately 10 cm, and the distance between the liquid surface and the receptor was approximately 15 cm. While the other conditions were fixed, aligned NFYs with different diameters could be obtained by varying the rotation speed of the receptor, ranging from 2 to 80 mm/min. The aligned NFY samples were collected from the receiver and kept at room temperature for at least 2 days before subsequent experiments.

### Morphological characterization and mechanical properties of aligned NFYs

Scanning electron microscopy (SEM, Sigma HD, United States) was used at an accelerating voltage of 2 kV to analyze the microstructure surface chemistry of aligned NFYs; each sample had three groups of replicates. The diameter distribution and alignment properties of nanofibers were counted using the ImageJ software module OrientationJ; each sample type had three replicates. Mechanical testing of single aligned NFYs was performed via dynamic thermo-mechanical analysis (Q800, DMA, United States). First, both ends of the single aligned NFY were clamped on filter paper and then fixed on the upper and lower arms of the instrument. The lengths of the aligned NFYs tested were approximately 2 cm. Uniaxial tensile testing was carried out at a stretching rate of 5 mm/min until specimen failure. As for the mechanical properties of bound NFYs, we bundled 50 NFYs of each diameter, and then used a universal testing machine (a series of LS, AMETEK, United States) to test the force and strain of these bound NFYs. The lengths of aligned NFYs tested were approximately 14 cm. Uniaxial tensile testing was carried out at a stretching rate of 5 mm/min until specimen failure. More than five parallel samples were measured for each sample of different NFY diameter.

### Isolation and culture of TSPCs

The Achilles tendon and patella tendon were obtained to isolate tendon stem/progenitor cells (TSPCs) from 6 to 8 week old Sprague Dawley (SD) rats, which were obtained following the approved guidelines set by the Institutional Animal Care and Use Committee, Southern Medical University. First, the peritendinous connective tissue was carefully removed, and the samples were washed three times with Dulbecco’s phosphate buffered saline (DPBS, Gibco, China). The samples were then minced into 2 mm^2^ pieces and digested with 3 mg/ml collagenase type I for 30 min at 37°C. Following digestion, the samples were diluted with DPBS, filtered through a 70 μm cell strainer, and then centrifuged at 1,000 rpm for 15 min. Single-cell suspensions were cultured in a cell culture incubator at 5% CO₂ and 37°C in Dulbecco’s Modified Eagle’s Medium (DMEM, Gibco, China) containing 20% fetal bovine serum (FBS, Gibco, Australia) and 1% penicillin/streptomycin. TSPC colonies were observed under a microscope after 5 days. A previous study has shown that TSPCs have good expression of tenomodulin (TNMD) up to passage 10 (Tan et al., 2012), so cells from passages 3-8 were used in our subsequent experiments.

### Characterization of TSPCs

Expression of stem cell surface markers was analyzed by flow cytometry. TSPCs (1.0 × 106 cells) in 500 μL of 10% FBS-DMEM were incubated with the following phycoerythrin-conjugated or fluorescein-isothiocyanate-conjugated mouse anti-rat monoclonal antibodies at 4°C for 1 h: anti-CD18 (561409, BD PHARMINGEN, United States), anti-CD34 (ab187284, abcam, United States), anti-CD44 (561409, BD PHARMINGEN, United States), anti-CD45 (MCA45PE, SEROTEC, United States), and anti-CD90 (561409, BD PHARMINGEN, United States). Before being tested, the cells were washed twice with DPBS and then re-suspended in 500 μL of ice-cold 10% FBS-DMEM and 1 μL propidium iodide. FACs results were analyzed using BD FACSCanto II (BD Biosciences, United States).

### TSPC culture on aligned NFYs

TSPCs were seeded and cultured on aligned NFYs of varying diameters following several steps. First, the aligned NFY samples were fixed around a 3D-printed rectangle model. Second, the aligned NFY samples were sterilized by immersion in 75% ethanol for 30 min, followed by 30 min of UV irradiation. After drying, the aligned NFYs were soaked in 1% ε-Poly-L-lysine solution for 30 min and dried overnight. Finally, the TSPC suspension (0.3 ml, 3×10^5^ cells/mL) was dropped onto aligned NFYs and allowed to adhere at 37° C for 4 h. When most cells had adhered to the surface of the NFYs, the aligned NFY samples were peeled off from the PDMS mold and placed into wells of a 6-well plate for continued culture for required experiments. The cell culture medium was changed every 2 days.

### Viability of TSPCs on aligned NFYs

The cellular activity of TSPCs seeded on the aligned NFYs was evaluated by Live/Dead assay (Beyotime, China) after 24 h and by Alamar Blue assay (Molecular Probes, Invitrogen, China) after 1, 5, and 7 days in culture, according to the manufacturer’s instructions. After being seeded on the aligned NFYs of various diameters, cells were cultured for 24 h to allow cell spreading. Cell-laden NFYs (*n* = 3) were then transferred to a new plate and washed with DPBS to remove the non-adherent cells. Calcein AM stained living cells with green fluorescence, and propidium iodide (PI) stained dead cells with red fluorescence. Before adding the working fluid, samples were washed with DPBS and then incubated for 30 min. Fluorescence imaging was carried out using confocal laser scanning microscopy (Nikon A1, China). Cells were incubated in 10% (v/v) Alamar Blue reagent in growth medium at 37° C for 4 h. The fluorescence of the supernatant was measured using a microplate reader (Synergy HT, Bio-Tek Instruments, United States) at an excitation wavelength of 560 nm and an emission wavelength of 590 nm.

### Morphological evaluation of TSPCs on aligned NFYs

The morphology and organization of TSPCs on aligned NFYs of various diameters were analyzed via immunofluorescence imaging after 3 days in culture; each sample had three replicates. Cultured cells were gently rinsed twice with DPBS for 3 min and then fixed with 4% PFA for 15 min at room temperature. After fixation, cells were gently rinsed with DPBS three times and then treated with 0.2% Triton X-100 for 45 min. The samples were blocked using 1% BSA for 2 h and then treated with fluorescein isothiocyanate (FITC)-phalloidin (Invitrogen, China) conjugated at 4° C overnight and counterstained with 4’, 6-diamidino-2-phenylindole (DAPI, Sigma, United States). Fluorescence imaging was carried out using a confocal laser microscope (Nikon A1, China). Images of F-actin fluorescence were processed using the OrientationJ module of ImageJ software and the elongation and orientation of TSPCs on these aligned NFYs were analyzed following the protocol of our previous study (Wang et al., 2015). Cell elongation was determined by measuring nuclear aspect ratios and the angle between the long axis of the cells and the direction of aligned NFYs to generate alignment histograms. Cells cultured on glass slides were the 2D control group.

### Tenogenic differentiation of TSPCs on aligned NFYs

TSPCs were cultured on aligned NFYs of various diameters in differentiation medium (growth medium with 50 mg/ml 2-phospho-L-ascorbic acid trisodium) to induce tenogenic differentiation for 7 days; each sample had three replicates. After 7 days of induction, the cell-seeded NFYs were carefully rinsed twice with DPBS and then fixed with 4% PFA for 15 min at room temperature. After fixation, the cells were carefully washed twice with DPBS and then treated with 0.2% Triton X-100 for 45 min. After blocking in 1% BSA in DPBS for 2 h, the cell constructs were incubated with rabbit anti-TNMD antibody (ab203676, abcam, China) and rabbit anti-collagen I (Col1a1) antibody (CO20191-01, Invitrogen, China) at 4° C overnight. The cell constructs were then washed with DPBS, and Alexa Fluor 488 (ab150077, abcam, China) conjugated secondary antibody was added and samples were incubated for 45 min at room temperature. After incubation, the cell constructs were washed with DPBS and cell nuclei were counterstained with DAPI for 5 min. Finally, the cell-seeded NFYs were observed under a confocal laser microscope (Nikon A1, China).

### Real-time qPCR

For real-time quantitative PCR (RT-qPCR) analyses, total RNA was extracted using Trizol (AG RNAex Pro Reagent, AG, China) following the manufacturer’s instructions. For synthesizing cDNA, 0.2–1 μL RNA was used in a reverse transcription reagents kit protocol (Abm, Canada). Three cDNA parallel compositions were obtained. Real-time qPCR was carried out according to the protocol. Briefly, qPCR reactions were first carried out on ice, and each well contained 7.5 μL MagicSYBR Mixture, 0.15 μL ROX, 0.5 μL cDNA, 6.35 μL H_2_O, and 0.6 μL mixed primers. The plates were inserted in an Applied Biosystems QuanStudio™^3^ Real-Time PCR (Singapore) Instrument for cycling at the following temperature profile: 95° C for 30 s, then 40 cycles of 95° C for 5 s followed by 60° C for 30 s. The primer sequences were as shown in Table 1. Each sample had three groups of replicates.

**TABLE 1:** Gene primer sequences for RT-qPCR.

Genes	5′-3′	Primer Sequences (5’→3′)	Production size (bp)
GAPDH	Forward	CTC​TCT​GCT​CCT​CCC​TGT​TC	105
	Reverse	TAC​GGC​CAA​ATC​CGT​TCA​CA	
TNMD	Forward	CAA​TGG​GTG​GTC​CCA​CAA​GT	231
	Reverse	TCG​ACC​TCC​TTG​GTA​GCA​GT	
DCN	Forward	ATC​ACA​GAA​GAG​GCA​ACG​AGC	163
	Reverse	GAG​ACT​TGC​GCC​AGA​AGG​AA	
Col1a1	Forward	ACG​CCA​TCA​AGG​TCT​ACT​GC	159
	Reverse	ACT​CGA​ACG​GGA​ATC​CAT​CG	
SCX	Forward	GAG​AAC​ACC​CAG​CCC​AAA​CA	84
	Reverse	CCG​TCT​TTC​TGT​CAC​GGT​CT	

### *In vivo* rat Achilles tendon repair model and histological evaluation

The animal experiment procedures were approved by the Institutional Animal Care and Use Committee, Southern Medical University. The SD rats (*n* = 6, 8-weeks-old) were treated with 1% pentobarbital sodium intraperitoneally at 40 mg/kg body weight. After anesthesia, a 6 mm length of the Achilles tendon was removed to make a defect model. Bundles of 50 yarns of NFY-50 were used to prepare the aligned NFY scaffolds to provide mechanical support for the repaired tendon. The NFY-50 scaffolds (50 yarns, length = 0.2 cm) were sutured to the residual Achilles tendon using a non-resorbable suture (5–0 nylon) via the Kessler method of tendon repair. Tendons that had been cut off directly served as the control group. The animals were allowed free movement after surgery. After 6 weeks of implantation, samples from each group were harvested and fixed in 4% PFA. Samples were then dehydrated through a series of increasing concentrations of ethanol, embedded in paraffin blocks, and sectioned at a thickness of 8μm. Sections were stained with hematoxylin-eosin (H&E), Masson’s trichrome, and Picrosirius red. Finally, the stained paraffin sections were observed under an optical microscope (Nikon Nxi, China). The Masson’s trichrome images were processed using the OrientationJ module of ImageJ software, with analysis of the orientation of neo-collagen in the defect site of each rat. Each group included three replicates.

### Statistical analysis

Experiments were run in triplicate for each sample and results are presented as mean ± standard deviation. Quantitative data and measurement of cell aspect ratios and orientation were obtained using ImageJ software, with results including at least 5 images of 3 independent locations within each sample (cell number >100). Statistical differences were obtained through analysis of variance followed by Tukey’s significant difference post hoc test. A significance level of 0.05 was applied to indicate significant differences.

## Results

### Preparation and Characterization of aligned NFYs

**FIGURE 2:**
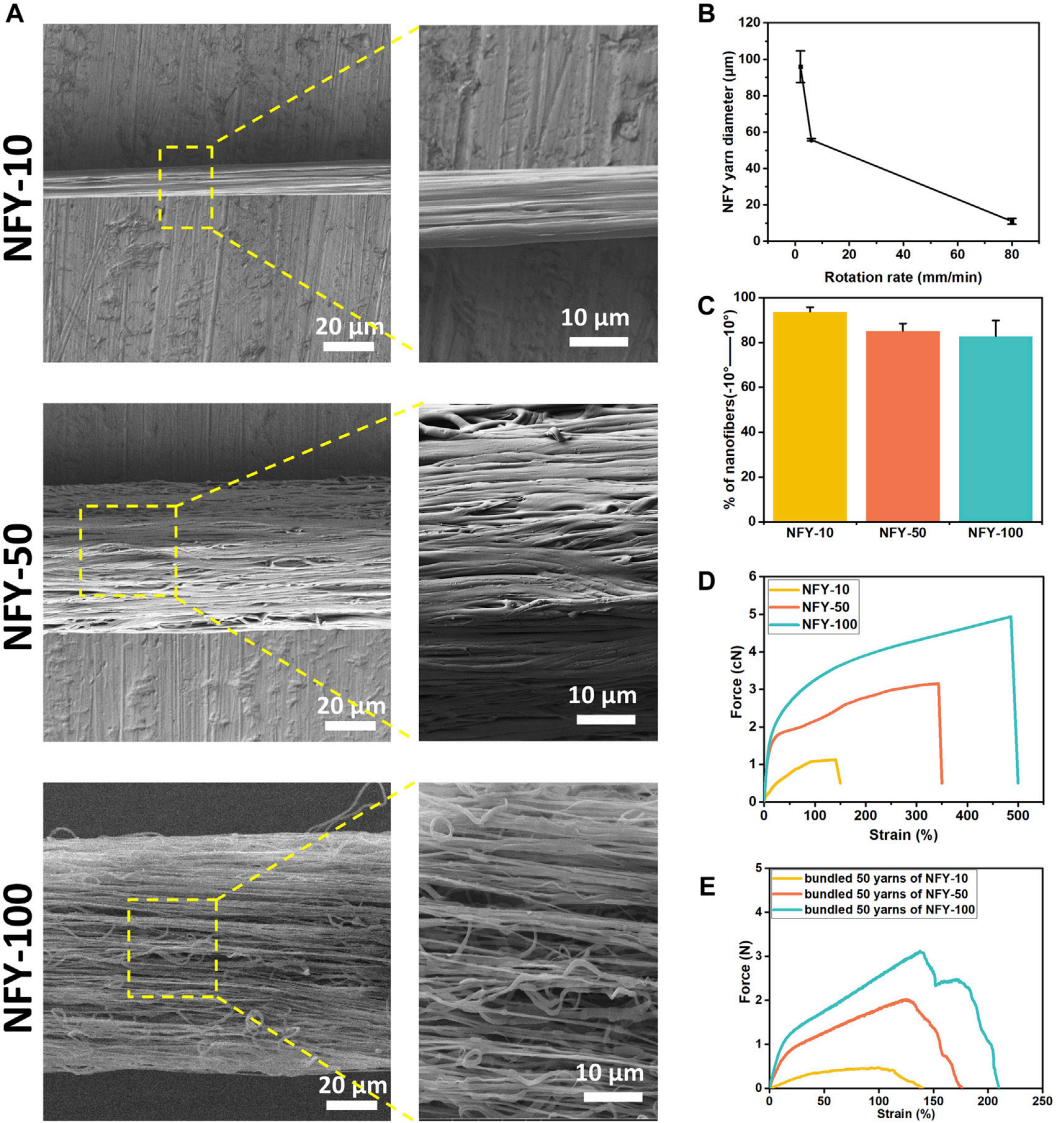
Microstructure and mechanical properties of aligned NFYs. **(A)** SEM images and their magnification images of aligned NFYs with average diameters of 10 μm, 50 μm, and 100 μm, respectively. **(B)** The relationship between and the diameter of NFYs and the rotation rate of the receptor. **(C)** Histograms of relative alignment within ±10^°^ show uniform orientation of nanofibers in aligned NFYs. **(D)** Force-Strain curves of NFYs with different diameters via single fiber tensile test. **(E)** Force-Strain curves of 50-yarn bundles of NFYs with different diameters via tensile test.

The fabrication of aligned NFYs based on a modified electrospinning device is shown in Figure 1B, and the process of fabricating these aligned NFYs is detailed in Supplementary Video S1. Briefly, the PCL polymer solution (100 mg/ml) was extruded by the pump in a high voltage field to prepare a mat of random nanofibers on the surface of a water/ethanol mixture. Subsequently, a rolling receptor pulled the nanofibers mat from the solution’s surface to form aligned NFYs. By controlling the rotation rate of the rolling receptor, a series of aligned NFYs with varying diameters were fabricated using the dry-wet electrospinning approach. The microstructure of the aligned NFYs was observed using SEM. The images showed that the aligned NFYs were loose and porous cylinders, and that the bundled NFYs had a hierarchical structure similar to native collagen fibers and fascicles of the tendon (Figure 2A; Supplementary Figure S1A). The nanofiber diameter was controlled, at 732.1 ± 167.22 nm by adjusting the parameters of electrospinning. The diameter of the aligned NFYs decreased dramatically from 100 ± 24 μm to 10 ± 2 μm when the receptor rotation rate increased from 2 to 80 mm/min, respectively (Figure 2B). The aligned NFYs with diameters of 10 ± 2 μm, 50 ± 10 μm, and 100 ± 24 μm were designated NFY-10, NFY-50, and NFY-100, respectively. NFY-10, NFY-50, and NFY-100 showed the proportion of oriented nanofibers, in the range of ±10^o^, to be 95.13%, 88.21%, and 85.66%, respectively (Figure 2C), suggesting that aligned NFYs have great improvement in axial orientation with increased rotation speed. The loaded-force of aligned NFYs was measured using a single fiber tensile test. The tensile analysis results showed that the force of a single NFY-100 was as high as 5.16 ± 0.04 cN, while NFY-50 was slightly decreased at 3.16 ± 0.16 cN, and NFY-10 was only 1.13 ± 0.13 cN (Figure 2D). Furthermore, the strain of aligned NFYs increased from 141 ± 41% to 486 ± 17% with the increase of NFY diameter from 10 µm to 100 μm, respectively. As shown in Figure 2E, the loaded-force of the bundled 50 yarns of NFY-10 was 0.44 ± 0.1 N with a stretching speed of 5 mm/min. Comparatively, the loaded-force of the bundled 50 yarns of NFY-100 was 3.31 ± 0.37 N. Notably, the loaded-force of the bundled 50 yarns of NFY-50 was 2.15 ± 0.09 N, which is suitable for tendon tissue with a loaded-force of 2–3 N (Table 2). These results revealed that nano- and micro-scale NFYs containing highly aligned nanofibers can be successfully prepared using the dry-wet electrospinning approach, with the mechanical properties of NFYs easily controlled by changing their diameters.

**TABLE 2:** Mechanical properties of single and 50-yarn bundles of aligned NFYs with different diameters.

Sample groups	Force	Strain
NFY-10	1.13 ± 0.13 cN	141 ± 41%
NFY-50	3.16 ± 0.16 cN	314 ± 16%
NFY-100	5.16 ± 0.04 cN	486 ± 17%
Bundled 50 yarns of NFY-10	0.44 ± 0.1 N	93 ± 18%
Bundled 50 yarns of NFY-50	2.15 ± 0.09 N	124 ± 25%
Bundled 50 yarns of NFY-100	3.31 ± 0.37 N	158 ± 26%

### Identification and characterization of TSPCs

**FIGURE 3:**
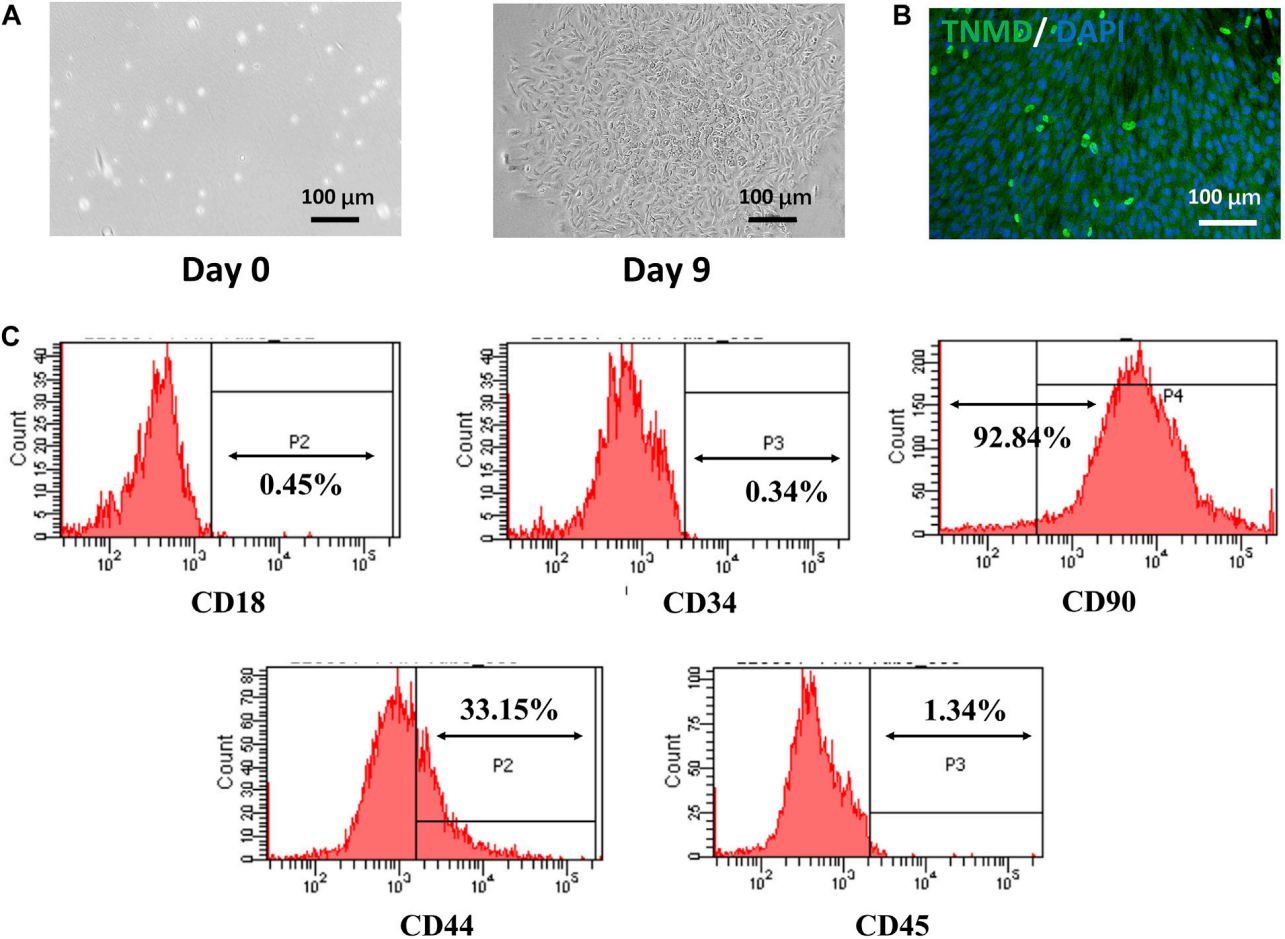
Isolation and Characterization of TSPCs. **(A)** The morphology of rat TSPCs at P0, **(B)** the tenogenic differentiation of TSPCs cultured for 7 days in differentiation medium, **(C)** Flow cytometry analysis of the expression of cell surface makers related to stem cells and hematopoietic cells.

After 3 days of isolation, a small number of TSPCs with spindle-like morphology were observed. The cells proliferated continuously and formed colony units after 9 days. The tenogenic differentiation capacity of TSPCs was verified by testing the expression of TNMD. As shown in Figure 3B, the TSPCs had obvious expression of TNMD. Furthermore, as shown in Figure 3C, over 92% and 33% of TSPCs were positive for BMSC markers CD90 and CD44, respectively, while they were negative for CD18, which is a surface receptor present on BMSCs (Miura et al., 2005). In addition, the TSPCs did not express hematopoietic cell markers, including CD34 and CD45, which are both highly expressed in tenocytes (Li et al., 2021), further confirming that these tendon-derived cells were TSPCs.

### Viability and morphology of TSPCs on aligned NFYs

**FIGURE 4:**
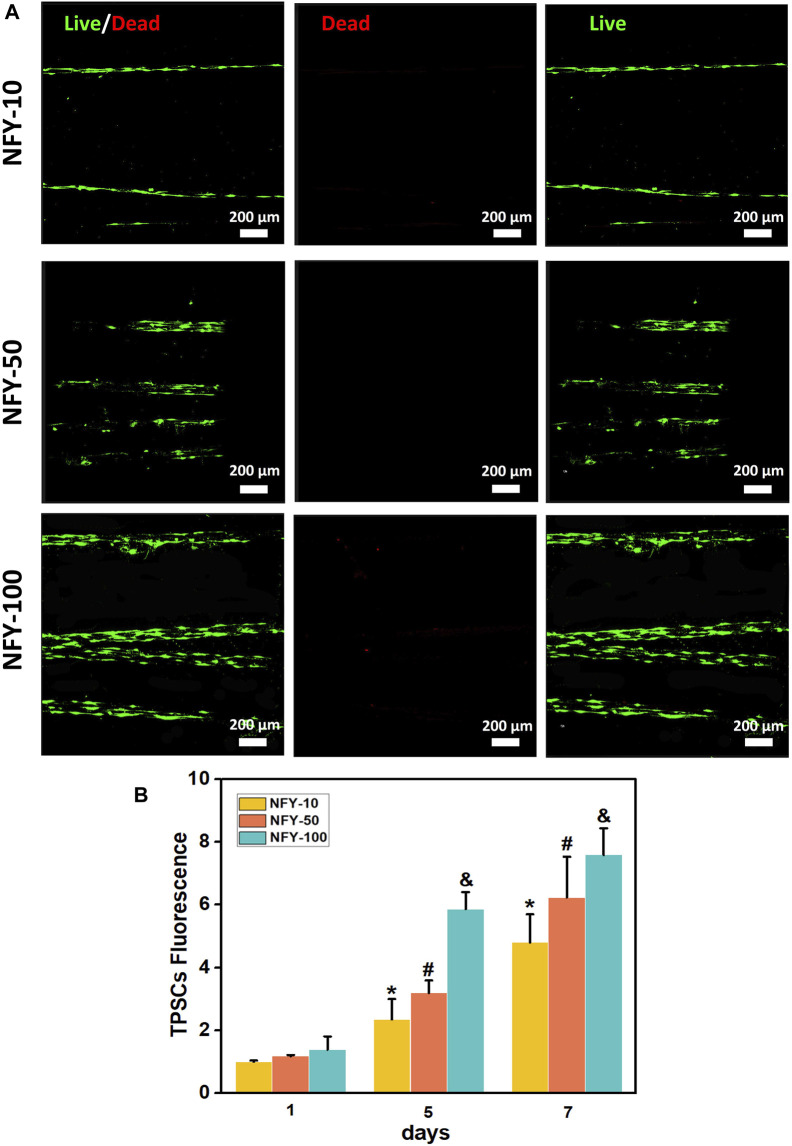
The viability of TSPCs on aligned NFYs with different diameters. **(A)** Fluorescence imaging of living (green) and dead (red) TSPCs seeded on aligned NFYs and cultured in growth medium for 24 h. **(B)** The proliferation of TSPCs on NFYs with different diameters, after 7 days, as measured using Alamar Blue. *p < 0.05 compared with the NFY-10 group at day 1, ^#^ *p* < 0.05 compared with the NFY-50 group at day 1, and ^&^ *p* < 0.05 compared with the NFY-100 group at day 1.

TSPCs were seeded on single aligned NFYs of various diameters, cell viability was assessed using the Live/Dead assay after for 24 h in culture, and cell proliferation was assessed using Alamar Blue reagent after 1, 5, and 7 days in culture. TSPCs cultured on aligned NFYs showed high viability, with few dead cells and excellent elongation (Figure 4A). The proliferation results showed that all aligned NFY groups had a significant increase in cell growth during the culture period. On the first day of cell culture, there were no significant differences between the aligned NFY groups (*p* > 0.05). As time in culture increased, the number of TSPCs on the aligned NFYs of various groups showed significant differences (*p* < 0.05). In particular, the NFY-100 group showed significantly higher cell numbers than the NFY-10 and NFY-50 groups on days 5 and 7 (Figure 4B). These results demonstrated that TSPC growth increases with increased NFY diameter, and the aligned NFYs, regardless of diameter, have good biocompatibility.

**FIGURE 5:**
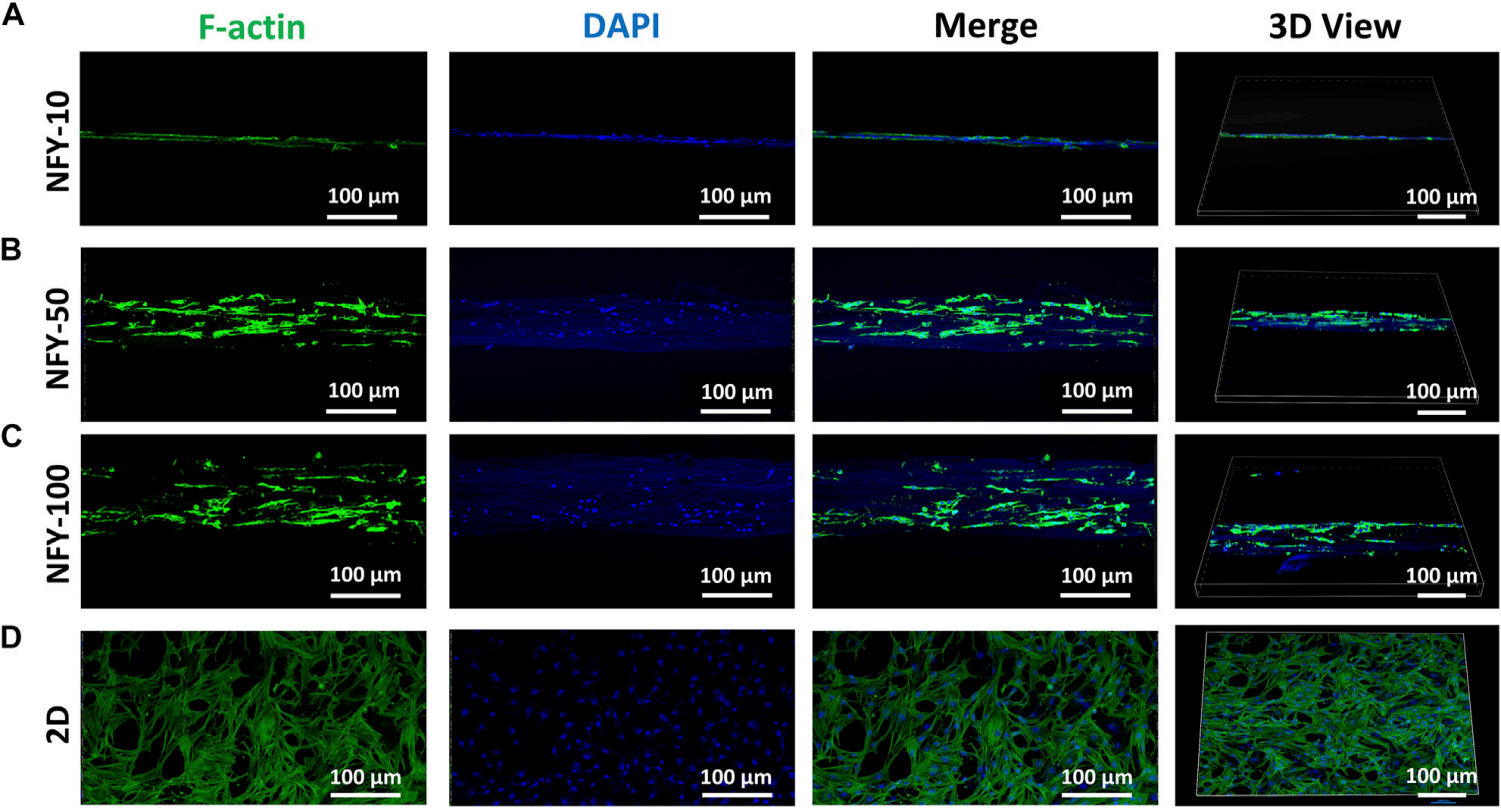
The top and 3D views of TSPCs with F-actin-stained cytoskeletons (green) and DAPI-stained nuclei (blue) after 3 days of culture on NFY-10 **(A)**, NFY-50 **(B)**, and NFY-100 **(C)**, as well as cells in the 2D group **(D)**. Scale bar = 100 μm. TSPCs cultured on single aligned NFYs with varying diameters showed aligned morphology, contrasting with the morphology of the 2D control group.

**FIGURE 6:**
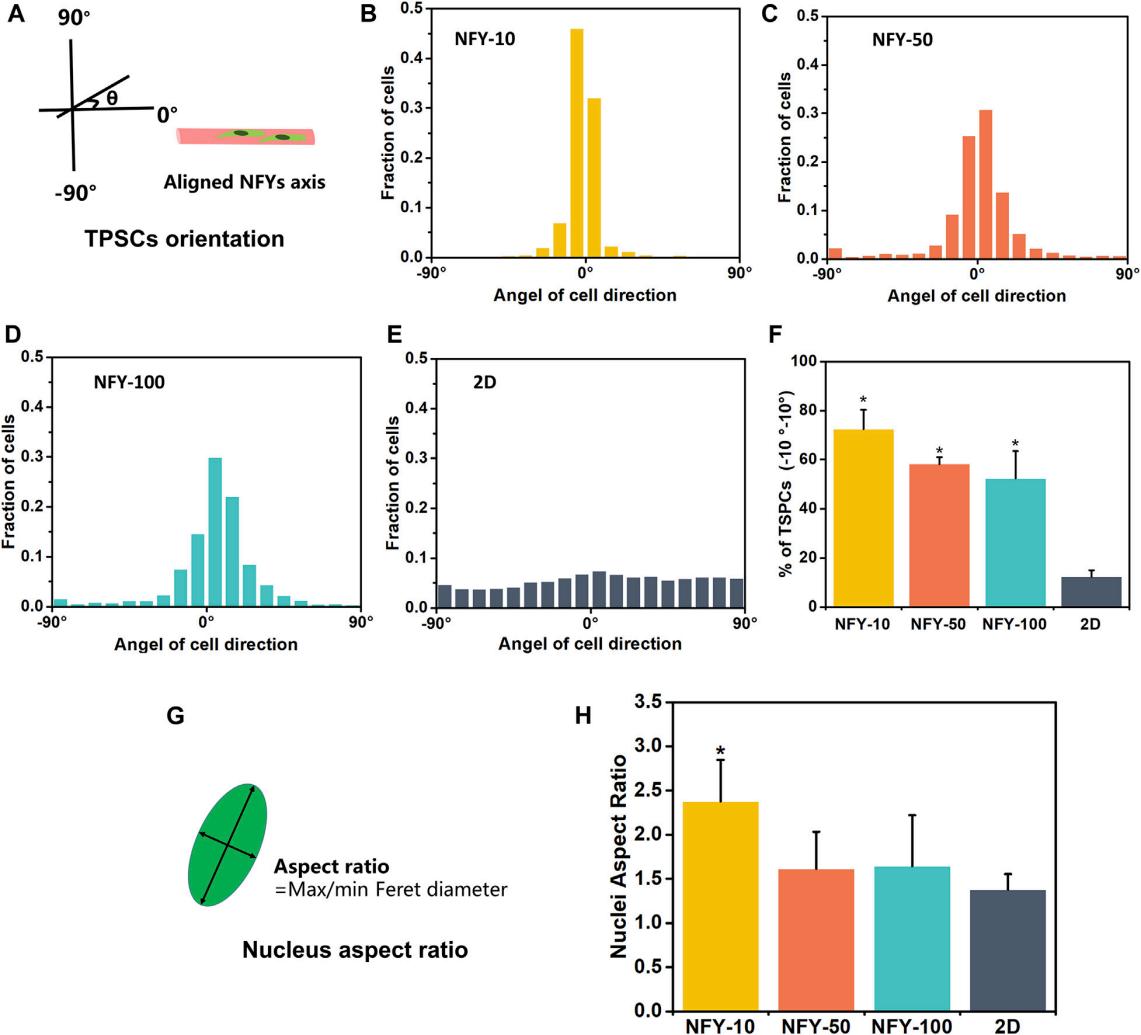
Quantitative measurement of cellular alignment and elongation of TSPCs on aligned NFYs. **(A)** Schematic diagram of cellular orientation measurement. The histograms show the cellular angles and distribution of TSPCs on NFYs with diameters of 10 μm **(B)**, 50 μm **(C)**, 10 μm **(D)**, and on a 2D tissue culture plate **(E)**. **(F)** Histograms of the relative alignment in ±10° increments demonstrate cellular alignment of TSPC on NFYs of varying diameters, in contrast to the random morphologies observed in the 2D group; *p < 0.05. **(G)** Schematic diagram of nuclear aspect ratios calculated from measurement of the length and width of the nucleus. **(H)** Histograms of nuclear aspect ratio of TSPCs on NFYs and TSPCs in the 2D group; *p < 0.05.

The cellular alignment and elongation behaviors of TSPCs on aligned NFYs with different diameters were assessed by F-actin staining after 3 days in culture. The cytoskeleton was stained green using FITC-conjugated phalloidin, and the nucleus was stained blue using DAPI. The images of F-actin staining show that TSPCs spread well on the surface of all aligned NFYs and exhibited aligned and elongated morphologies (Figures 5A–C). In contrast, cells in the 2D group showed random spindle morphology (Figure 5D). Furthermore, quantitative analysis of cell alignment was accomplished by measuring the angle between cell growth direction and the direction of aligned NFYs, including relative alignment in ±10° increments (Figure 6A). These data show that the orientation ratio of cells within ±10^o^ on NFY-10, NFY-50, and NFY-100 were 73%, 58%, and 53%, respectively (Figures 6B–E), while the orientation ratio was only 12% in the 2D group (Figure 6F). Additionally, cell elongation behavior was estimated by assessing TSPC nuclear aspect ratios, which demonstrated a significant improvement in cellular elongation of the NFY-10 group compared to that of the 2D group (Figure 6H). Together, these data indicate that aligned NFYs are able to enhance the 3D cellular alignment and elongation of TSPCs, which mimics native collagen fibers with tenocyte alignment. Notably, TSPCs showed better alignment and elongation behaviors when cultured on aligned NFYs with smaller diameters.

### Tenogenic differentiation of TSPCs on aligned NFYs

**FIGURE 7:**
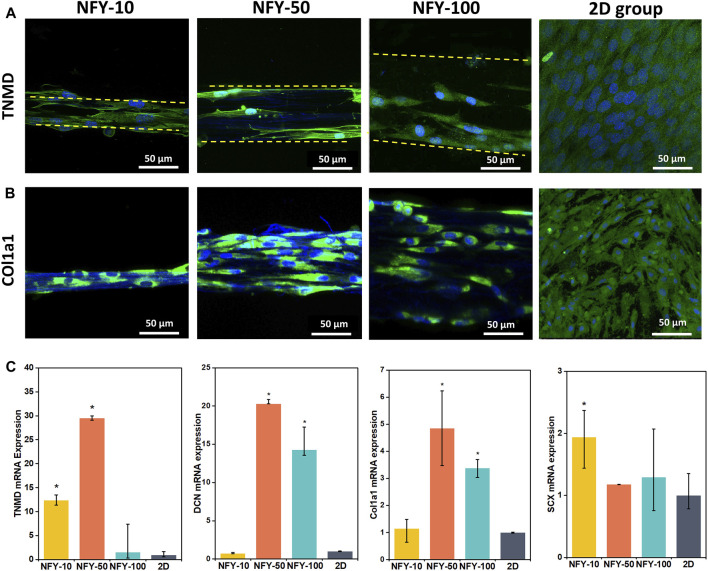
Tenogenic differentiation of TSPCs cultured on aligned NFYs of varying diameters. **(A)** Immunofluorescence images of TNMD staining of TSPCs on aligned NFYs of different diameters, and cells from the 2D control group, after 7 days in culture; green indicates TNMD, blue indicates cell nucleus. **(B)** Immunofluorescence images of Col1a1 staining of TSPCs on aligned NFYs of different diameters, and cells from the 2D control group after 7 days in culture; green indicates Col1a1, blue indicates cell nucleus. **(C)** RT-qPCR analysis of tenogenic gene expression in TSPCs cultured on aligned NFYs and cells from the 2D group. (*n* = 3; *p < 0.05).

To explore the effects of aligned NFYs on tenogenic differentiation of TSPCs, cells were seeded on aligned NFYs of various diameters and then cultured in differentiation medium for 7 days. TNMD is a marker of the mature tenogenic phenotype. Type Ⅰ collagen (Col1a1) is a major matrix component of native tendons that supports essential mechanical properties of tendon tissue (Goh et al., 2014). Tenogenic differentiation was examined by TNMD immunostaining. Images of TNMD and Col1a1 staining showed that the fluorescence intensity in all of the aligned NFY groups was higher than that of the 2D group, indicating that TSPCs on these aligned NFYs had higher TNMD and Col1a1 expression compared with the 2D group, and that aligned NFYs enhanced tenogenic differentiation of TSPCs, as suggested by enhanced expression of these markers (Figures 7A,B). Additionally, NFY-50 showed the highest TNMD expression, compared with NFY-10 and NFY-100, which indicates that the best tenogenic differentiation of TSPCs occurred on NFY-50.

We also investigated the effects of cell culture on aligned NFYs on the tenogenic differentiation of TSPCs maintained in growth medium for 7 days. Decorin (DCN) is the most abundant small protein-polysaccharide rich in leucine in the tendon and plays a key role in ensuring precise alignment and stability of collagen fibers related to the phenotype of the tendon. Scleraxis (SCX) is an essential transcription factor for tenogenesis (Bavin et al., 2017). RT-qPCR results showed that aligned NFYs improve the expression of tendon-related genes, including TNMD, DCN, Col1a1, and SCX (Figure 7C). Gene expression of TNMD and DCN was approximately 30 times and 20 times greater, respectively, in cultures with NFY-50 compared with the 2D group (*p* < 0.05). These results indicate that the 3D aligned and multi-scale structure of aligned NFYs may improve the tenogenic differentiation of TSPCs. Interestingly, cells cultured with NFY-50 showed the highest expression of TNMD, DCN, and Col1a1. These results are consistent with the TNMD and Col1a1 immunofluorescence staining results, further confirming the enhanced effect of aligned NFYs on tenogenic differentiation of TSPCs.

### Aligned NFYs aid in repair of rat Achilles tendon injury *in vivo*

**FIGURE 8:**
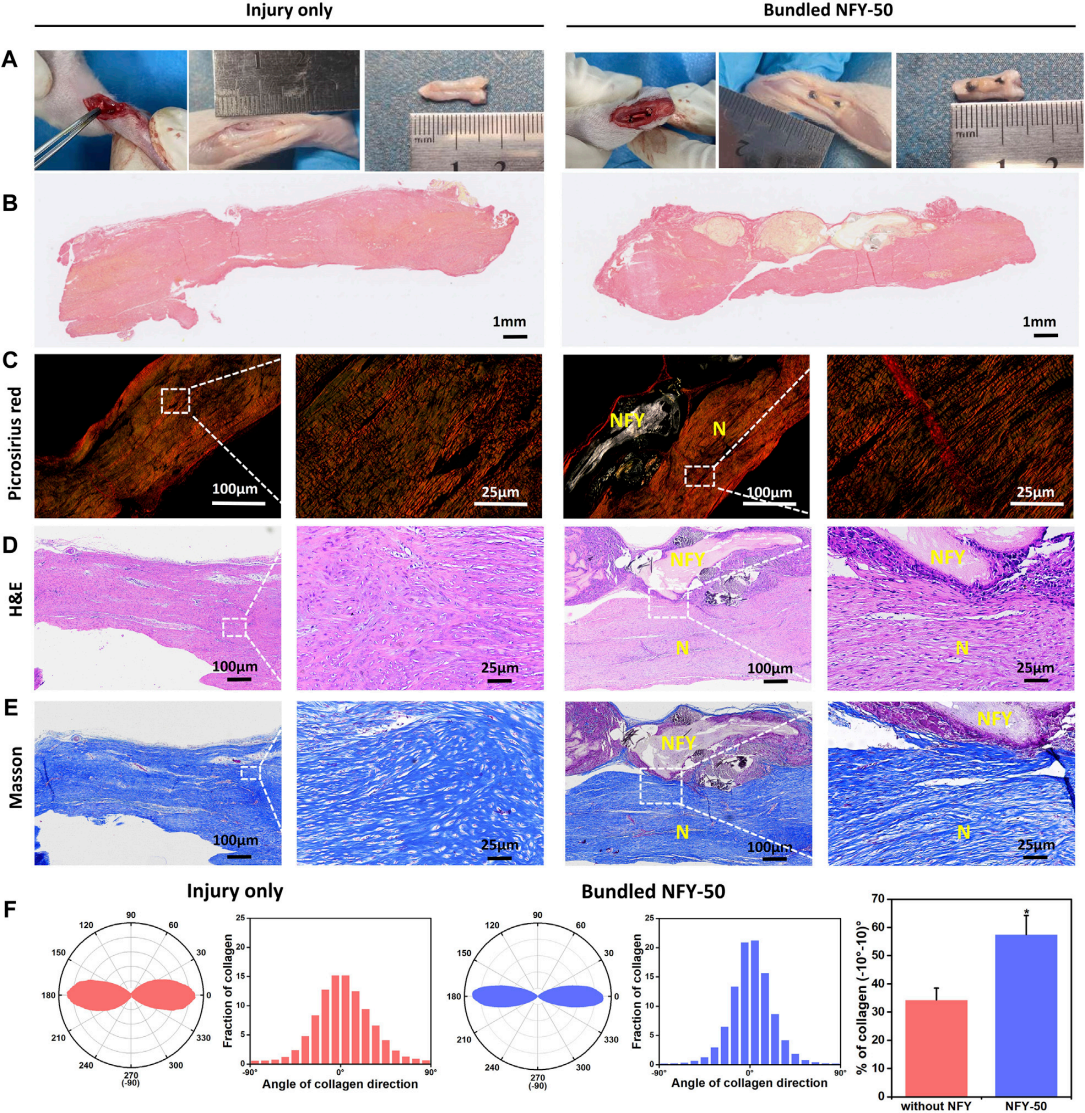
The effects of aligned NFYs on tendon repair *in vivo*. **(A)** NFY bundles were implanted into the rat Achilles tendon injury model; tendons were cut off directly in the injury only group. **(B)** Whole Picrosirius red staining with a light mirror. **(C)** Picrosirius red staining with a polariscope. **(D)** Images of H&E and **(E)** Masson’s trichrome staining of the defect site 6 weeks after bundled NFY implantation or injury only treatment. For Picrosirius red staining, red indicates collagen I and green indicates collagen Ⅲ. N, neo-collagen; NFY, NFY-50. **(F)** Quantitative analysis of collagen orientation and distribution and histograms showing relative alignment in ±10° increments. *p < 0.05.

The ability of single NFYs to improve the tenogenic differentiation of TSPCs has been verified. Therefore, to investigate the effects of NFYs on improving tendon regeneration *in vivo*, we implanted 50-yarn NFY bundles into a rat tendon injury model. The NFY-50 group showed significantly better tenogenic differentiation of TSPCs compared with the other NFY groups. Therefore, we used NFY-50 to further investigate effects of NFYs on *in vivo* tendon repair. Tendon injury usually includes three sequential phases from injury onset to healing: the injured tendon initially becomes inflamed, forming granulation tissue within the injury region to serve as a provisional matrix. Then, type III collagen fibers, fibronectin, and proteoglycans are formed at the injury site and type III collagen fibers are replaced by type Ⅰ collagen fibers at the proliferation stage. Finally, there is alignment of tenocytes and collagen fibers, with type Ⅰ collagen fibers increasing and type III collagen fibers decreasing (Vasiliadis and Katakalos, 2020). In our study, 6 weeks after implantation, partial neo-tissue with a typical white, tendon-like appearance was observed in rats that received the bundled NFY-50 scaffold (Figure 8A). To identify the type of collagen prominent in the neo-tissue, we used Picrosirius red staining to distinguish collagen types in the tendon defect area. As shown in Figure 8C, the aligned NFY scaffold group formed more collagen I (red), with little collagen Ⅲ (green) remaining; in contrast, the injury only group showed more collagen Ⅲ formation, indicating that aligned NFY scaffolds improve tendon regeneration. H&E staining results (Figure 8D) showed partial formation of aligned neo-tissue around the NFY-50 scaffold. Bundled NFY-50 scaffolds surrounded by high cell nuclei density indicated that the nanofiber structure of NFY-50 had allowed immune cells and blood infiltration, which may have enhanced tissue remodeling. Images taken at high magnification showed partial neo-tissue that was organized and aligned, as it was guided by the aligned micro- and macro-structure and mechanical support of the bundled NFY-50 scaffold. In contrast, the neo-tissue in the injury only group was disordered and showed typical scar tissue without aligned structure. Masson’s trichrome staining (Figure 8E) results also indicated that neo-tissue formation included an abundance of collagen fibers (blue), and showed that the collagen fibers had an aligned microstructure along the direction of the NFY-50 scaffold. These quantitative data demonstrate that the orientation ratio of collagen within ±10°in the injury only and NFY-50 groups were 34% ± 4% and 57% ± 6%, respectively. Therefore, the injury only group showed amorphous organization, indicating fibrous scar tissue formation, compared to alignment in the NFY-50 group.

## Discussion

Native tendon tissues show a hierarchical structure, where highly-aligned collagen fibers form fascicles, and many fascicles are bound together to form the tendon (Goh et al., 2014). Tenocytes, in particular, lay on the longitudinally aligned collagen fascicles and fibers and exhibit cellular orientation. This hierarchical structure plays a key role in the high tensile strength of native tendon. Therefore, developing a biomimetic TE scaffold with a hierarchical structure that enhanced cellular alignment, elongation, and mechanical strength would improve tendon repair (Vasiliadis and Katakalos, 2020). In preparing the biomimetic scaffold, the first challenge is in providing suitable mechanical support for the newly synthesized ECM at the injury site during tendon tissue regeneration. In this study, we chose PCL polymer as the primary material from which to develop the electrospun NFY due to its suitable mechanical properties, good biocompatibility, and broad use in various biomedical applications (Sun et al., 2006; Hu et al., 2016).

The electrospinning technique is regarded as a promising approach to preparing aligned nanofibers. For instance, previous studies have shown that aligned nanofiber sheets promote cellular elongation and tenogenic differentiation (Watari et al., 2012; Tu et al., 2020). However, those nanofiber sheets have failed to mimic the complex hierarchical structure of native tendon tissue. In contrast, we have reported on a series of NFYs, prepared using a well-developed dry-wet electrospinning technique, that were able to guide 3D cellular alignment and elongation in soft TE applications (Wu et al., 2017b; Wang et al., 2019). Therefore, in this study, we utilized our dry-wet electrospinning system to prepare the aligned PCL NFYs with an abundance of aligned nanofibers that mimicked the hierarchical structure of alignment fascicles and collagen fibers in native tendon tissue (Figure 2; Supplementary Figure S1). The aligned NFYs with smaller diameters had better alignment structure but less robust mechanical properties (Figure 2D). The rotation rate of the NFY receptor played a key role in the diameter and orientation of NFYs. When the receptor was rotating faster, the random nanofibers would be dragged from the surface of the solution and received on the receptor faster. Therefore, the NFYs would have better alignment structure and smaller diameter with increased rotation rate of the receptor. In addition, a previous study suggested that nanofiber orientation was affected by the rate of the receptor with only one variable (Vimal et al., 2016). The mechanical properties of murine tendons showed 2–3 N according to a previous study (Kurtaliaj et al., 2019). In comparison, we found that the 50-yarn bundles of NFY-50 had suitable force with 2.15 ± 0.09 N, demonstrating suitable mechanical properties for rat tendon tissue repair compared with NFYs of other diameters.

Several types of stem cells isolated from different tissue sources, such as bone marrow, adipose tissue, and tendon, have been applied in tendon TE applications (Bi et al., 2007; Bavin et al., 2015; Wu et al., 2017a; Laranjeira et al., 2017). Among them, TSPCs, which are isolated from tendon sources and differentiate into tenocytes to regulate tendon homeostasis, would be more suitable for the tendon microenvironment (Guo et al., 2016). A previous study revealed promising results from the use of TSPCs in tendon injury repair *in vivo* (Youngstrom et al., 2016). In our study, we used TSPCs to evaluate the effects of aligned NFYs on tenogenic differentiation *in vitro*, after successful isolation of TSPCs from rat tendon tissue and confirmatory assessment of tenogenic markers using flow cytometry (Figure 3). The composition and structure of the ECM regulates the phenotype and differentiation of TSPCs and affects the regeneration of functional tendon tissue (Sheng et al., 2019). Recent studies have investigated the effects of nanofiber diameters on the behavior of tendon cells (English et al., 2015; Jenkins and Little, 2019). However, there are still few reports on investigation of scaffolds with integrated hierarchical structure that induce TSPCs behavior and phenotypes that lead to tenogenic differentiation. In our study, we successfully prepared aligned NFYs of varying diameters, allowing us to study the effects of NFY diameter on cellular response and tenogenic differentiation of TSPCs. The proliferation results showed that the TSPCs on NFY-50 and NFY-100 exhibited higher proliferation rates compared with the other groups (Figure 4). One of reasons for this phenomenon was greater contact inhibition on NFYs of smaller diameter due to reduced area available for cell growth. In a prior study, Gerard and Goldbeter (2014) created a detailed computational model to interpret contact inhibition, which suggested that the proliferation of cells would be inhibited by the Hippo/YAP pathway rather than culture conditions when cell density reached the threshold. Another reason was higher initial adhesion rate in the groups with NFYs of larger diameters, with the larger diameter providing increased adhesion area and greater area for cell growth. As observed by the results of Alamar Blue staining on day 1 in culture, there was a rising trend in the number of TSPCs on the NFYs with increasing diameters. Moreover, our previous study showed that cell adhesion would be limited by the narrower surface of the aligned NFYs of smaller diameter, which is consistent with the results of the current study. On the other hand, F-actin staining confirmed that the 3D aligned NFYs provided a 3D microenvironment that induced TSPCs to orient in alignment with the NFYs (Figures 5, 6). More importantly, recent studies demonstrated that nanofiber scale could affect cell differentiation (Sheng et al., 2019). For instance, Bashur et al. (2009) showed that electrospun nanofibers exhibited nanoscale fiber diameters similar to those of collagen fibers in native tendon, and that the nanofibers could increase the level of TNMD gene expression. In our study, both RT-qPCR and immunofluorescence staining to assess levels of the tenogenic markers TNMD, DCN, and Col1a1 showed that use of NFYs of 50 μm diameter significantly improved the tenogenic differentiation of TSPCs compared with use of aligned NFYs of other sizes (Figure 7). These results are mainly due to the aligned structure of NFY-50 and it’s diameter, which is similar to the collagen fascicles and fibers of rat tendon tissue of approximately 50–300 μm in diameter. Ultimately, aligned NFYs with 3D hierarchical structure consisting of aligned monodisperse nanofibers promoted tenogenic differentiation of TSPCs.

To further investigate the effects of the aligned NFYs on tendon repair *in vivo*, NFY-50 was chosen for implantation due its superior ability to promote TSPCs tenogenic differentiation and suitable mechanical properties. We bundled 50 yarns of NFY-50 together to prepare the aligned NFY scaffolds to mimic the macrostructure of the tendon tissue and provide mechanical support for the repaired tendon. We then made a defect in the Achilles tendon of a rat model and implanted the scaffold. Tissue was harvested 6 weeks later to obtain sections for H&E, Masson’s trichrome, and Picrosirius red staining. NFY-50 scaffolds resulted in better histological structure and regeneration ability compared with the injury only group (Figure 8). Based on histological evaluation, there was obvious degradation of aligned NFYs due to the greater surface area of the nano-scale structure (Zhang et al., 2014). A previous study showed that the PCL grafts composed of nanofiber sheets had undergone significant degradation, suggesting an advanced cell response when the tendon was repaired during the 6-week period of remodeling (Bosworth, 2014). In our study, NFYs composed of numerous nanofibers showed a higher degradation rate and many macrophages gathered around the scaffold. The main reason for inflammation was the adsorption of protein on the surface of implanted NFY scaffolds due to the nanofibrous structure (Zhou and Groth, 2018). With degradation of the bundled NFY-50, neo-collagen filled in the scaffolds after 6 weeks. The complete tissue sections (Picrosirius red staining) are shown in Figure 8B, demonstrating that the bundled NFY-50 connected the fracture of the tendon as a bridge, and that neo-collagen appeared inside the degraded area of the bundled NFY-50 to wrap it externally. Upon degradation of the bundled NFY-50, i.e., the PCL materials, neo-collagen replaced the NFY scaffold to achieve repair of the tendon.

To further distinguish different types of collagen, we used polarized light microscopy (PLM) to image tissue with Picrosirius red staining, where type Ⅰ collagen fibers appeared bright red-yellow, while type Ⅲ collagen fibers appeared green (Rittié, 2017). A recent study revealed that type Ⅰ collagen fibers are associated with mechanical strength (Goh et al., 2014). The type III collagen fibers would be gradually replaced by type I collagen fibers during tendon remodeling, and type I collagen fibers begin to align along the long axis of the tendon (Deng et al., 2014). In this study, the Picrosirius red assay demonstrated that the type Ⅰ collagen fibers were stained bright red-yellow in the NFY scaffold group in contrast with the injury only group, indicating that the bundles of NFY-50 improved mechanical strength and induced neo-collagen alignment of the neo-tendon tissue. There were fewer type Ⅲ collagen fibers in the aligned NFY scaffolds than in the injury only group (Figure 7D). These results indicate that the aligned NFYs enhance regeneration of tendon tissue and, at the same time, guide the aligned formation of collagen fibers during tendon repair.

Above all, this study confirmed that PCL 3D aligned NFYs prepared by dry-wet electrospinning provide suitable mechanical support and the hierarchical structure guidance for tendon tissue regeneration, and that NFY-50 improves tenogenic differentiation of TSPCs as a biomimetic of similar diameter to collagen fascicles and fibers in native tendon. Bundled NFY scaffolds mimic the macrostructure of tendon tissue and provide mechanical support for the repaired tendon *in vivo*. There is high potential for use of aligned NFY-50 in development of additional scaffolds that require element units to produce hierarchical 3D aligned scaffolds for the construction of tissue-specific functional architecture, thus advancing regenerative therapies for soft tissue repair.

## Conclusion

In this study, highly aligned NFYs of varying diameters were fabricated and characterized to investigate their effects on the behavior of TSPCs. We found that aligned NFYs could mimic the topography and mechanical properties of collagen fascicles and fibers of native tendon. Aligned NFYs induced TSPCs orientation and elongation. Moreover, aligned NFYs enhanced tenogenic gene and protein expression, including that of TNMD; NFYs of 50 μm diameter showed the greatest effect. Taken together, these data indicate that the nanofibrous structure of aligned NFYs is able to induce TSPCs orientation and elongation, and the muti-scale structure of aligned NFYs may improve tenogenic differentiation. To further investigate the effects of aligned NFYs on tendon repair *in vivo*, 50-yarn bundles of NFY-50 were implanted in rat Achilles tendon models, with the results demonstrating that the aligned hierarchical NFY scaffolds induced neo-collagen orientation and cell alignment*,* and that bundled NFY-50 scaffolds enhanced tendon tissue regeneration. Overall, these results suggest that aligned NFYs of well-defined diameter mimic the complex hierarchical structure of native collagen fascicles and fibers and show excellent potential for use in tendon regeneration applications.

## Data Availability

The raw data supporting the conclusions of this article will be made available by the authors, without undue reservation.
