# Supplementary material for: Electrospun aligned poly(ε-caprolactone) nanofiber yarns guiding 3D organization of tendon stem/progenitor cells in tenogenic differentiation and tendon repair
Source: Front Bioeng Biotechnol. 2022 Aug 30;10:960694. doi: 10.3389/fbioe.2022.960694 (PMC9468671; doi:10.3389/fbioe.2022.960694)
Supplement: Supplementary file 1 [file Table1.DOCX]

# Supporting information

**Electrospun aligned poly(ε-caprolactone) nanofiber yarns guiding tendon stem/progenitor cells 3D organization for tenogenic differentiation and tendon repair**

Qiao Yang^1^, Jianfeng Li^1,3^, Weiwei Su^2^, Liu Yu^2^, Ting Li^2^, Yongdi Wang^3^, Kairui Zhang ^4^, Yaobin Wu^2^*, Ling Wang^1^*

^1^Biomaterials Research Center, School of Biomedical Engineering, Southern Medical University, Guangzhou, 510515, P.R. China

^2^Guangdong Engineering Research Center for Translation of Medical 3D Printing Application, Guangdong Provincial Key Laboratory of Medical Biomechanics, National Key Discipline of Human Anatomy, School of Basic Medical Sciences, Southern Medical University, Guangzhou 510515, P.R. China

^3^The First School of Clinical Medicine, Southern Medical University, Guangzhou, 510515, China

^4^ Division of Orthopaedics and Traumatology, Department of Orthopaedics, Nanfang Hospital, Southern Medical University, Guangzhou, 510515, China

***Correspondence:** Ling Wang [wangling0607@smu.edu.cn](mailto:wangling0607@smu.edu.cn);

Yaobin Wu wuyaobin2018@smu.edu.cn


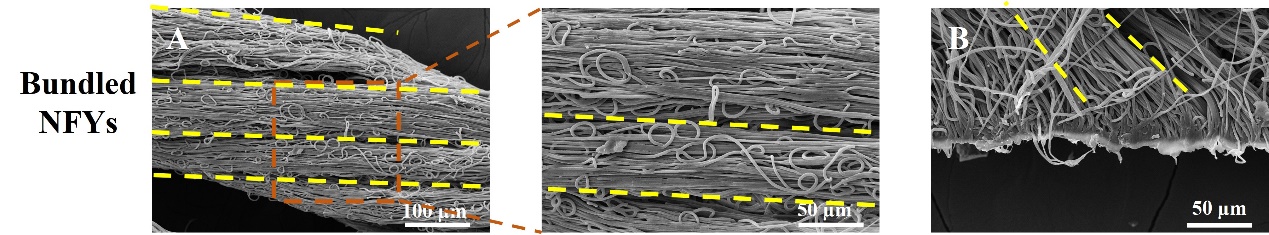


Figure S1 The microstructure properties of bundled NFYs. (A) The horizontal for the bundled NFYs SEM image. (B) The vertical for the bundled NFYs SEM image. The orientation of these aligned NFYs was marked by these yellow wines.

Movie S1 The process of fabricating these aligned NFYs.
